# The Effect of the Substrate on the Microstructure and Tribological Properties of Cold Sprayed (Cr_3_C_2_-25(Ni20Cr))-(Ni-graphite) Cermet Coatings

**DOI:** 10.3390/ma15030994

**Published:** 2022-01-27

**Authors:** Anna Trelka, Wojciech Żórawski, Łukasz Maj, Paweł Petrzak, Dominika Soboń, Anna Góral

**Affiliations:** 1 Institute of Metallurgy and Materials Science, Polish Academy of Sciences, 30-059 Krakow, Poland; l.maj@imim.pl (Ł.M.); p.petrzak@imim.pl (P.P.); 2 Faculty of Mechatronics and Mechanical Engineering, Kielce University of Technology, 25-314 Kielce, Poland; ktrwz@tu.kielce.pl (W.Ż.); dsobon@tu.kielce.pl (D.S.)

**Keywords:** cold spraying, cermet coating, microstructure, wear resistance

## Abstract

In this work, the effect of the substrate, Al 7075 alloy and 1H18NT9 stainless steel, on the microstructure and tribological properties of cold sprayed (Cr_3_C_2_-25(Ni20Cr))-(Ni-graphite) coatings was investigated. Both coatings were dense and did not reveal any discontinuities at the interfaces. They had similar Cr_3_C_2_ and graphite contents. Their microstructures showed a variety of grain sizes of the matrix phase between the inner part of the splat, showing large ones, and their boundaries, where elongated and nanostructured grains were formed during the deposition process. The coating deposited on the steel substrate revealed a slightly higher hardness and lower abrasive wear with the Al_2_O_3_ loose abrasive particles. The force required to destroy the durability of the coating–steel substrate system in the three-point bending test was higher than those of the other ones. The cermet deposit cold sprayed on steel and examined at 25 °C under 10 N revealed the best wear resistance and the lowest friction coefficient.

## 1. Introduction

Composite coatings are commonly used to protect working elements, mainly in the printing, automotive and aviation industries, where wear occurs in the processes of intense friction at high temperatures. Cermet coatings containing ceramic particles embedded in a metallic matrix meet criteria such as anti-wear, high hardness, and compact microstructure with good adhesion [1,2,3,4]. Protective Cr_3_C_2_-NiCr deposits are manufactured mainly using plasma spraying and HVOF; however, cold spraying is increasingly becoming popular [5,6,7,8,9,10,11,12,13,14,15,16,17,18,19,20]. This type of coating has been deposited on various substrates, usually on low-carbon steel [5,6,7,8], corrosion-resistant steel, and aluminium alloys [21], depending on the application of the working element. Compared to other deposition techniques, cold spraying ensures more advantages of the obtained coatings, for example, no phase transformation and delamination, and low porosity. Cold sprayed deposits are produced by strong plastic deformation of the metal particles at a temperature below the melting point of the feedstock material. The metal powder particles undergo an adiabatic shear instability and form a coating by mechanical interlocking into the substrate [10,11,12,13]. Ceramic particles embed into a metal/alloy matrix. The use of ceramics in the coating material allows the properties of the coatings to be improved. The literature data describing cold sprayed cermet Cr_3_C_2_-NiCr coatings show that, depending on the used substrates, they reveal various amounts of Cr_3_C_2_ embedded in their structure and mechanical properties [14,15,16,17,18,19,20,21,22]. For cold sprayed Cr_3_C_2_-25(Ni20Cr), the carbide content built in the deposit was in the range of 22.8–33.8 vol.%, whereas the hardness changed from 449 HV0.3 to 875.7 HV0.2. The above results show that the substrate used has a lower impact on the microstructure, but significantly changes the mechanical properties of the deposited coatings. However, it should be noted that these authors used different powder types with different morphologies. As was shown, the Cr_3_C_2_-25(Ni20Cr) deposits cold sprayed from Oerlikon Diamalloy 3004 powder on the Superni 75 superalloy revealed a higher hardness (875.67 HV0.2) [22] than those sprayed on the substrate made of Al 7075 (635 HV0.3) [17]. It follows that various parameters of coating production and the use of other cold spray systems affect the quality of the deposits produced. Yin et al. [23] showed that the substrate of cold sprayed Ti coatings influenced the thickness of the obtained deposits. They revealed that the deposits on substrates with greater hardness, such as steel, were almost half as thin as those sprayed on Cu and Al, and there was better bonding of the Ti particles in cold spraying. However, no research has so far studied the systematic impact of the substrate on the microstructure, mechanical and tribological properties of the Cr_3_C_2_-NiCr coatings with graphite obtained from the same powder and under the same cold spray process conditions, and they are a novelty. In terms of determining the effect of the substrate on the coating thickness and microstructure, the deposition of only metal particles on various substrates has been studied so far. The cold spraying process allows coatings with the same phase composition as the feedstock powder to be deposited. The graphite could be introduced into the deposit this way. Furthermore, the addition of a solid lubricant into Cr_3_C_2_-25(Ni20Cr) powders allows cermet coatings to be produced with a dense microstructure and good mechanical properties, reducing the wear under conditions of severe friction.

This work aimed to investigate the influence of the substrate (Al 7075 alloy and 1H18NT9 stainless steel) on the microstructure, mechanical, and tribological properties of cold sprayed (Cr_3_C_2_-25(Ni20Cr))-(Ni-graphite) cermet coatings. The substrate used determines the application of these coating–substrate systems. The (Cr_3_C_2_-25(Ni20Cr))-(Ni-graphite) coatings cold sprayed on Al 7075 alloy can be used where a low weight of the elements is needed, while those deposited on steel can operate at higher temperatures. They possess self-lubricating properties and improve the anti-wear properties of substrates. So far, there is limited data on Cr_3_C_2_-25(Ni20Cr) deposits containing graphite as a solid lubricant obtained in the cold spray process [18,19]. Therefore, a detailed examination of these coatings will fill the knowledge gap in this field and show how the used substrates affect the microstructure and properties of these cermet deposits.

## 2. Materials and Methods

The (Cr_3_C_2_-25(Ni20Cr))-(Ni-graphite) coatings were deposited using a mixture of two commercial powders: 95 wt.% Cr_3_C_2_-25(Ni20Cr) Diamalloy 3004 and 5 wt.% Ni25C Durabrade 2221, manufactured by Oerlikon-Metco GmbH, Immelborn, Germany. The deposits were sprayed onto two substrates: Al 7075 alloy and 1H18N9T steel. The chemical compositions (wt.%) of the Cr_3_C_2_-25(Ni20Cr) and Ni-25C powders, as well as Al 7075 alloy and 1H18N9T stainless-steel substrates, are presented in Table 1. The substrate was a flat bar with dimensions of 400 mm × 30 mm × 5 mm. Prior to deposition, its surface was grid-blasted with F30 electro-corundum (600–710 µm). The cold spray process was performed by employing an Impact Innovations 5/8 cold spray system, mounted on a Fanuc M-20iA robot arm using the cold spray process parameters shown in Table 2.

**Figure 1:**
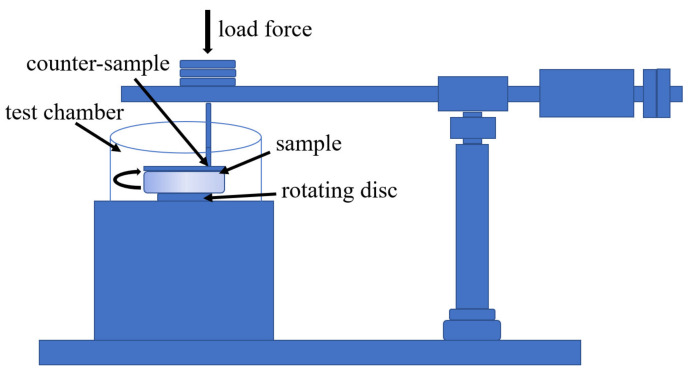
A scheme of the ball-on-disc device.

Phase analysis of powders and coatings was carried out using a Bruker D8 Discover X-ray diffractometer (Bruker AXS GmbH, Karlsruhe, Germany) with CoKα radiation (wavelength of 1.7903 Å), Diffrac.EVA V3.0, and HighScore Plus 4.8 software with the ICDD PDF-4+ crystallographic database. The surface morphology and microstructure of coating cross-sections were visualised on a micro-scale using an FEI E-SEM XL 30 scanning electron microscope (FEI Company, Hillsboro, OR, USA). The content of the ceramic phase and the graphite in the coatings was determined using image analysis (ImageJ) based on cross-section microstructures (10 images in the case of each deposit) obtained using SEM. The transmission electron microscopy (TEM) investigations were performed on a nano-scale using an FEI Tecnai G2 SuperTWIN 200 kV microscope (FEI Company, Oregon, United States) equipped with an SIS MegaView III CCD camera for the acquisition of the microstructure images in bright-field mode and the recording of selected-area electron diffraction patterns (SAED). The analysis of chemical composition was performed with an energy-dispersive X-ray spectrometer produced by EDAX. Phase analysis was conducted using CSpot V1.1.0 and Carine V3 computer software. The TEM samples were cut-out, applying the focused ion beam (FIB) technique, a ThermoFisher Scios 2 Dual Beam microscope (Thermo Fisher Scientific Inc., Waltham, MA, USA) and EasyLift lift-out system. The thin foils were attached to the copper grid using the Pt gas injection system. The surface topography of the as-sprayed, unpolished coatings was tested by a Profilm 3D profilometer (Filmetrics/KLA Corporation, San Diego, CA, USA). The 1500 µm × 2000 µm maps showing the topography of deposits with geometric parameters measured according to ISO 25178 [26] were presented. The low-load Vickers hardness HV0.3 was measured ten times on the cross-section of the deposited coatings with a microhardness tester from CSM Instruments (Anton Paar GmbH, Graz, Austria), and the average value was presented. The coating hardness was measured in accordance with the ISO 6507-1:2018(EN) standard [27]. The abrasive wear tests with loose abrasive particles (Al_2_O_3_ particles in the size range 250–300 µm) were performed using an ITEE T-07 tester (The Institute for Sustainable Technologies, Radom, Poland) (dry sand-rubber wheel) under a load of 50 N at a flow rate of 250 g/min and a wheel (Ø50 × 20) rotation speed of 200 rpm. Tests of the friction coefficient and the wear index of coatings were carried out with the use of a ball-on-disc T-21 tribotester (The Institute for Sustainable Technologies, Radom, Poland), with its scheme presented in Figure 1. The ball was a 6 mm-diameter sintered Si_3_N_4_ sphere, its linear sliding speed was 0.1 ms^−1^, the radii were 5, 7, and 8.5 mm, and the number of cycles was 20,000. The investigations were performed under two loads of 10 N and 20 N and at two temperatures of 25 °C and 250 °C for deposits sprayed on the Al 7075 substrate, and under two loads of 10 N and 20 N and three temperatures of 25 °C, 250 °C, and 500 °C for coatings deposited on the steel substrate. The friction and wear characteristics were tested in accordance with the ISO 20808:2016(E) standard [28]. The surface of the coating was ground and polished on diamond polishing suspensions with a finishing gradation of 1 µm. Thus, all samples had a rough surface roughness (Ra) equal to 0.016 ÷ 0.018 µm.

The wear tracks after the tribological tests were visualised using an optical microscope (Leica Microsystems Wetzlar GmbH, Wetzlar, Germany). The three-point bending test was performed in pursuance of the ISO 7438:2016(E) standard [29] at room temperature on an INSTRON 6025 modernised by Zwick/Roell (Ulm, Germany) with a computer-controlled mandrel traverse speed equipped with a system for a three-point bending test. The specimens were cut from the coating–substrate system, ground on abrasive papers, and polished on diamond suspensions to obtain the size of 1.2 mm × 3 mm × 24 mm. To eliminate the influence of the deposit or substrate thickness on the obtained results, both had the same thickness. The samples were bent at a speed of 0.001 mm·s^−1^ until the coating cracked. The former moving into the substrate–coating system with a constant speed of 0.001 mm/s^−1^ caused an increase in applied force. The investigations were performed for three samples of each system. The measurement of the coating adhesion to the substrate was made with a PosiTest AT using the pull-off method (ISO 4624:2016 [30]) by pulling the dolly stuck to the deposit with a hydraulic actuator. The PosiTest AT smoothly increased the pull-off force until the maximum value, occurring just before the sample detachment from the substrate, was recorded. The result was an average value from the three measurements.

## 3. Results and Discussion

### 3.1. Powder Characterisation

**Figure 2:**
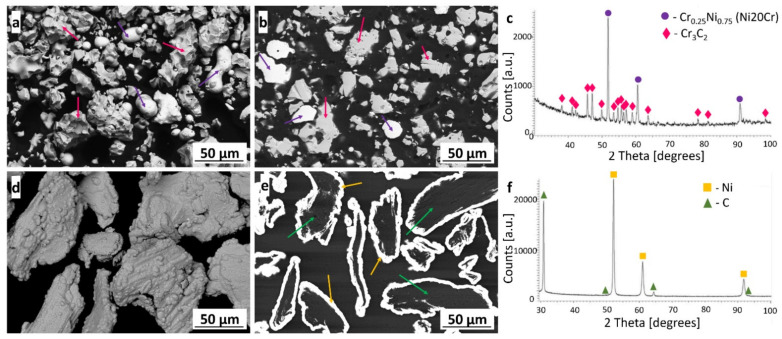
The morphology, cross-section, and XRD phase analysis of the Cr_3_C_2_-25(Ni20Cr) (**a**–**c**) and Ni25C (**d**–**f**) powders.

The (Cr_3_C_2_-25(Ni20Cr))-(Ni25C) was sprayed from a mixture of two powders in the ratio 95 wt.% to 5 wt.% of (Cr_3_C_2_-25(Ni20Cr)) and Ni25C. The Diamalloy 3004 powder consisted of irregular Cr_3_C_2_ ceramic particles and a spherical-like Ni20Cr matrix powder (Figure 2a,b). The Ni25C powder consisted of graphite (solid lubricant) particles coated by nickel (Figure 2d,e). The X-ray diffraction phase analysis of the powders revealed the phase composition given by the manufacturer. The following phases were observed: Cr_0.25_Ni_0.75_, Cr_3_C_2_ [16], Ni, and C (Figure 2c,f).

### 3.2. Microstructure Characterisation of Coatings

**Figure 3:**
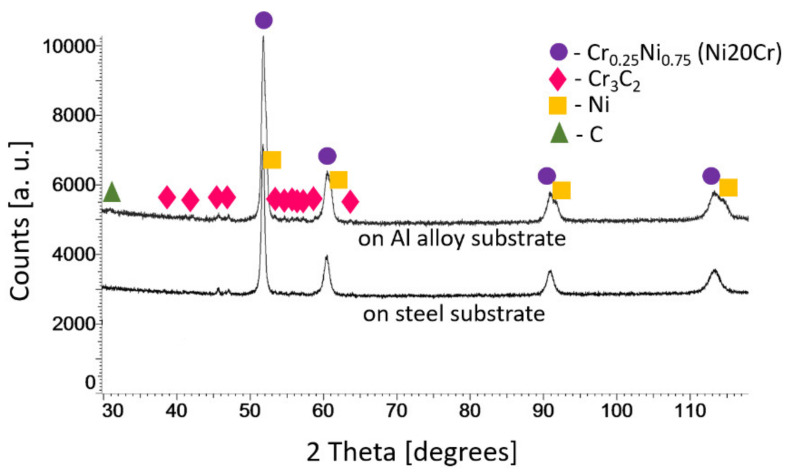
X-ray diffraction patterns for the (Cr_3_C_2_-25(Ni20Cr))-(Ni25C) coatings deposited on the Al 7075 alloy and 1H18N9T steel substrates.

The X-ray diffraction phase analysis of the (Cr_3_C_2_-25(Ni20Cr))-(Ni25C) coatings deposited on the Al 7075 alloy and 1H18N9T stainless-steel substrate showed the same phases that were identified in the feedstock powders (Figure 3). XRD patterns did not show significant differences in phase peak intensities, though a more intense peak from graphite was observed in the coating on the Al 7075 substrate, which corresponds to its higher vol.% content.

The substrate material affected the deposit thickness of 1190 ± 23 µm and 960 ± 19 µm (an average from ten measurements) for the Al alloy and steel substrates, respectively. 

**Figure 4:**
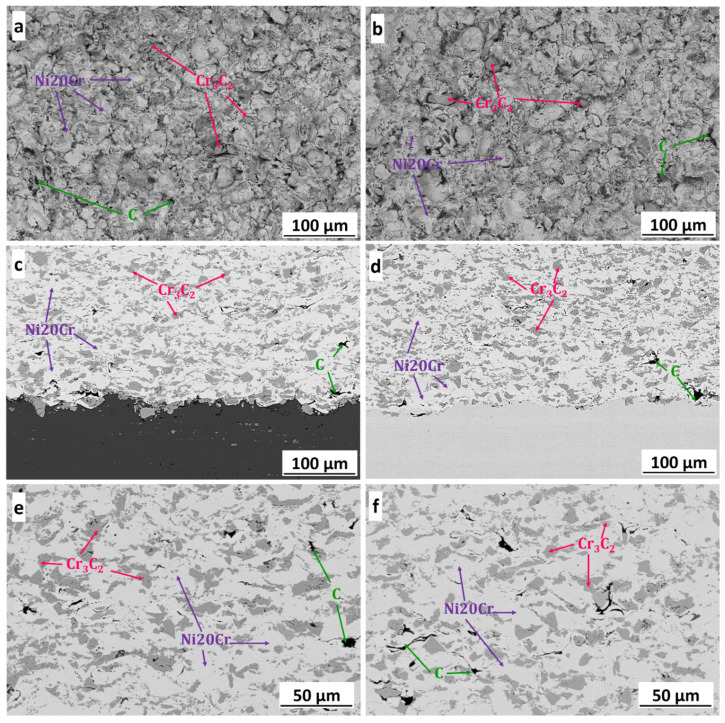
SEM surface morphologies and cross-sections of the (Cr_3_C_2_-25(Ni20Cr))-(Ni25C) coatings deposited on the Al 7075 alloy (**a**,**c**,**e**) and steel (**b**,**d**,**f**) substrates.

Figure 4 shows the surface morphology and cross-section microstructure of coldsprayed (Cr_3_C_2_-25(Ni20Cr))-(Ni25C) coatings deposited on the Al alloy (Figure 4a,b) and steel substrates (Figure 4c,d). The morphology of the coatings showed Cr_3_C_2_ particles and graphite embedded in the Ni20Cr matrix. The cross-section of the coatings showed an evenly distributed carbide phase in the matrix and graphite mainly located near the ceramic particles, as well as between severely deformed splats of the alloying phase. A compact microstructure with no visible porosity was observed regardless of the substrate used. The absence of discontinuities in deposits confirmed the good adhesion of the coatings to the substrates. The above-indicated microstructure features of (Cr_3_C_2_-25(Ni20Cr))-5(Ni25C) composite coatings were observed in the papers of Góral et al. [16] and Żórawski et al. [19], who described in detail the cermet deposit formation during the cold spray process. Image analysis performed using ImageJ software showed that the coatings deposited on the Al alloy substrate had 31.5 ± 1.9 vol.% Cr_3_C_2_ and 1.6 ± 0.4 vol.% C, while the coatings deposited on the steel substrate had 32.3 ± 1.3 vol.% Cr_3_C_2_ and 1.3 ± 0.2 vol.% C in their structure. During cold spraying, the initial stage of deposit formation is related to the activation of the substrate caused by an adiabatic shear instability and the associated localisation of the plastic flow at the interface, which plays a critical role in ensuring particle cleanliness and substrate contact surfaces. The ceramic particles impacting the substrate or previously deposited layer are crushed and bounced; therefore, there is less ceramic phase in the coatings produced than in the feedstock powder [16,17]. The volume percentage of ceramic and graphite phases was similar in the coating deposited on different substrates and was within the standard deviation range. This proves that the use of different substrates does not significantly affect the volume percent of Cr_3_C_2_ built in the deposits. However, the coatings sprayed on the Al alloy substrate showed more ceramic particles stuck in the substrate than those on the steel. They were also greater and embedded deeper than in the second deposit (Figure 4c,d; Figures 6b,d and 9a,b). This effect was directly related to the hardness of these substrates. The 1H18N9T steel substrate was 25% harder (220 HV1) than the Al 7075 substrate (177 HV1). The difference in hardness meant the deposited material was more easily embedded in the softer Al alloy substrate. The first Cr_3_C_2_ particles deposited well in the softer Al alloy, where the phenomenon of mechanical interlocking takes place, while subsequent carbide particles significantly fragmented as a result of greater particle crushing. In turn, ceramic falling on the steel substrate crumbled and bounced, consequently causing the substrate to bond mainly with a Ni20Cr phase via the adiabatic shear instability process, and a smaller amount of finely divided Cr_3_C_2_ carbides were embedded. It should be emphasised that the Cr_3_C_2_ particles were brittle, and their high presence in the interfacial zone weakened the bond between the coating and the Al alloy substrate. This significantly affected the adhesion of the deposits to the substrate. This was confirmed by the results of the coating adhesion to the substrate measured with the PosiTest AT, which were 34.7 ± 0.7 MPa and 40.6 ± 3.0 MPa for the deposits sprayed on the Al alloy and steel substrates, respectively. 

**Figure 5:**
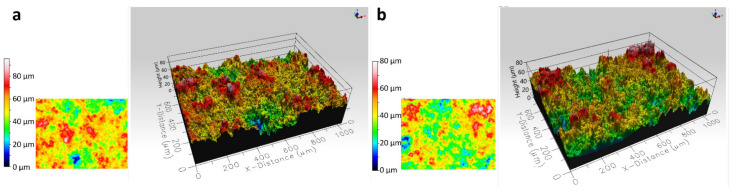
Surface topography of the (Cr_3_C_2_-25(Ni20Cr))-5(Ni25C) coatings: on the Al 7075 alloy (**a**), on the 1H18N9T steel (**b**).

Table 3 shows the geometric parameters of the surfaces measured according to ISO 25178 [26] based on the surface topography maps (Figure 5). The obtained results show that the use of different substrates did not affect the surface topography of the coatings. The values of the parameters arithmetic mean height (S_a_), root-mean-square height (S_q_), and skewness (S_sk_) were similar regardless of the substrate used. The coatings had a surface with random roughness and the S_sk_ was close to zero. The kurtosis (S_ku_) parameter was 3.06 and 2.67 for the coatings deposited on the Al alloy and steel substrate, respectively. This indicated that the surfaces of these coatings were free from extreme peaks or valley features. The other S_p_, S_v_, and S_z_ parameters calculated from the absolute highest and lowest points were higher for the deposit sprayed on the Al alloy. 

**Figure 6:**
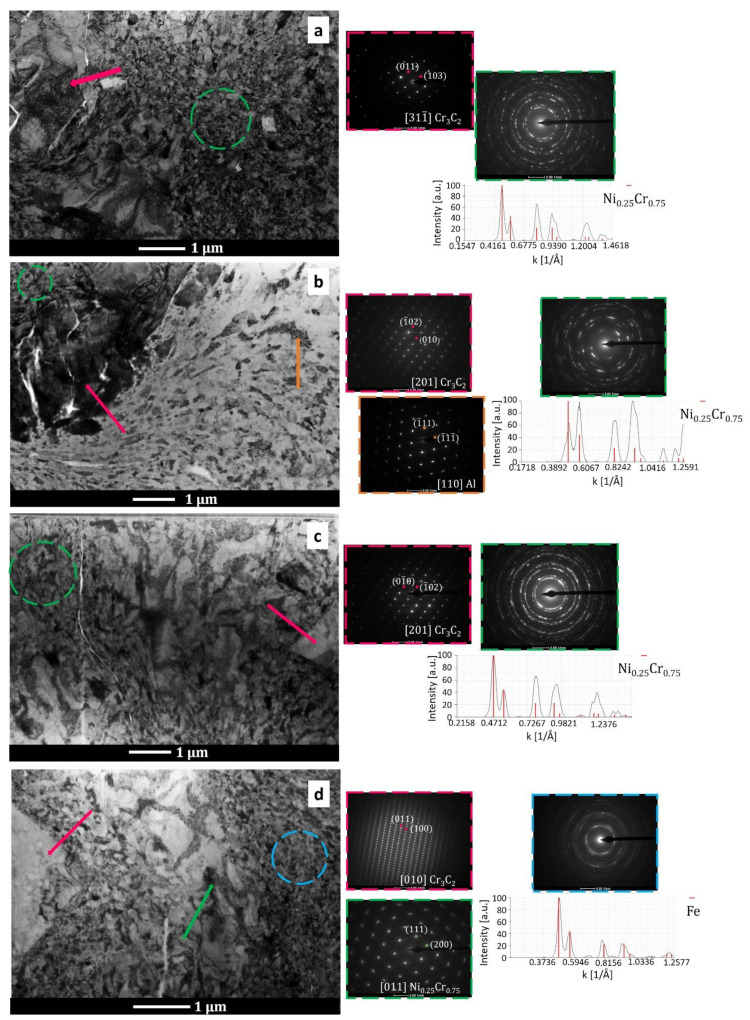
Bright-field TEM images and selected-area diffraction patterns in the areas showing the coating structure in the central part of the coating sprayed on the Al alloy (**a**), near the Al substrate (**b**), in the central part of the coating sprayed on steel (**c**), and near the steel substrate (**d**).

**Figure 7:**
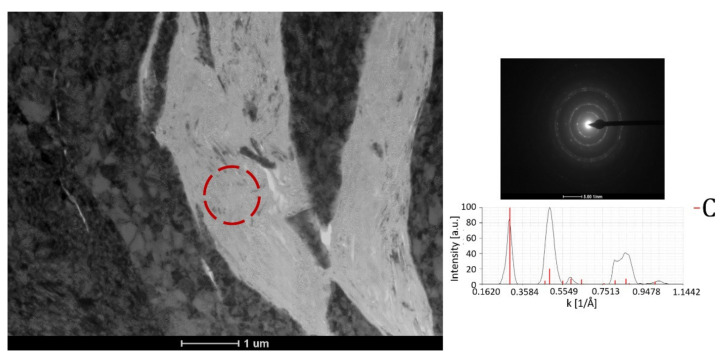
Bright-field TEM image of the graphite with selected area diffraction pattern.

**Figure 8:**
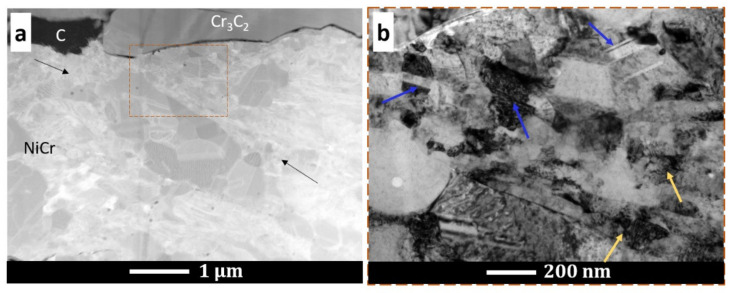
STEM microstructure of the cermet coating with visible matrix grain refinement (black arrows) (**a**) and BF microstructure with the nanotwins (blue arrows) and dislocation accumulation (yellow arrows) (**b**).

**Figure 9:**
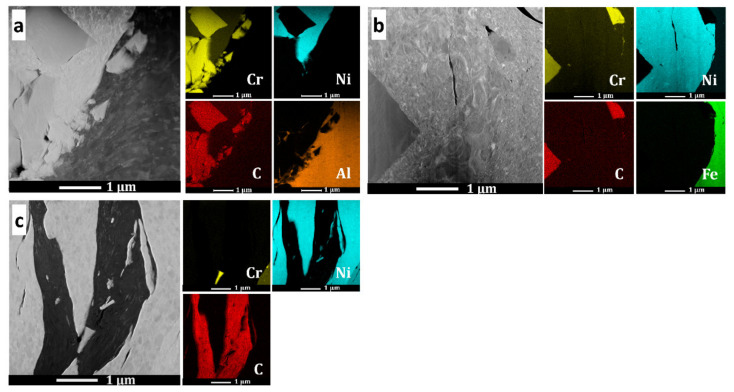
STEM microstructures and chemical element distribution maps in the coatings in the area near the Al alloy substrate (**a**), steel substrate (**b**), and graphite (**c**).

Figure 6 shows the TEM bright-field microstructure and selected-area electron diffraction patterns of the (Cr_3_C_2_-25(Ni20Cr))-(Ni25C) coatings and deposit–substrate interfaces for the applied substrates, i.e., Al 7075 alloy (Figure 6a,b) and 1H18N9T steel (Figure 6c,d). Both cold sprayed coatings were dense and did not reveal any discontinuities at the interfaces. They were composed of strongly deformed grains of Ni20Cr forming splats, between which Cr_3_C_2_ particles were embedded, and the graphite in elongated forms was surrounded by a thin shell of nickel, which was as expected (Figure 2e and Figure 7). The graphite had a fibre structure and the Ni phase consisted of very small grains. The Ni20Cr phase was both in the form of elongated grains and finely divided grains, especially in the areas near the ceramic particles, at the interfaces of the deformed particle–particle and the particle–substrate, Figure 6, Figure 8 and Figure 9. Ni20Cr grains in the vicinity of the interface region were slightly refined relative to the grains in the inner region of the deposited particles. In the centre of the splat, the matrix grain size was much larger than in the surrounding area because its inner part did not experience too much plastic deformation. The differentiation in the grain size of the matrix phase is particularly noticeable in Figure 8 and Figure 9. In the cold sprayed cermet coatings, the matrix phase underwent extensive high-strain-rate plastic deformation, and grains were highly deformed and elongated. Nanograins or elongated grains formed by the adiabatic shear instability impacted with high-velocity alloy particles. This phenomenon was limited to a periphery part of the particle–particle and particle–substrate interface [31]. In these areas, there was a high density of defects, particularly shear bands, as shown in Figure 6, which were the direct consequence of adiabatically induced localised plastic flow along these boundaries. The adiabatic shear instability in the interfacial strongly deformed regions may also result in the temperature rising rapidly to cause dynamic recrystallisation by lattice and subgrain rotation, as was shown by Zou et al. and Yin et al. [32,33]. Chaudhuri et al. [34], who investigated the cold sprayed Inconel 625 super alloys on a steel substrate, showed a ~50% coating grain size reduction in comparison with the particle size of the feedstock powder. They showed that the supersonic velocity of the deposit particles impacting the substrate caused their significant deformation and thermally activated dynamic recrystallisation at the interfacial region. The fine-grained microstructure in the vicinity of the substrate–coating interface was the effect of the stored plastic strain energy associated with dislocations, which increased with the strain rate [34]. 

Ultra-high strain rate and strain with a low deformation temperature promoted deformation twinning. It was shown in Figure 8 that the numerous twins and nanotwins were generated by deformation twinning in the forms of elongated ultrafine grains along Ni20Cr grains. This agrees with the examinations reported by Bae et al. [35] in cold sprayed Ni coatings. The twins significantly lowered the size of matrix grains and improved the deposits’ mechanical properties.

As can be seen in Figure 6b,d, the coatings were well bonded with the substrates. Figure 6b presents the interface zone where the Cr_3_C_2_ particle was stuck in the Al alloy substrate. The fine-grained Ni20Cr matrix adhered well to the ceramic phase. This ceramic particle underwent mechanical interlocking in the substrate. In this area, the Al alloy grains were significantly refined, as an effect of local deformation strengthening by the strong impact of feedstock powder particles. Similar grain refinement was observed in the case of the steel substrate (Figure 6d). The analysis of the selected-area diffraction patterns (Figure 6) confirmed the occurrence of the same phases, as shown by XRD investigations. Figure 9 shows the maps of the chemical element distribution made at the coating–substrate interfaces and in the area of localised graphite. The EDS examinations revealed the presence of Cr, Ni, and C in the coatings, and Al and Fe in the analysed substrates.

### 3.3. Mechanical and Tribological Properties of Coatings

#### 3.3.1. Hardness of the Deposits

**Figure 10:**
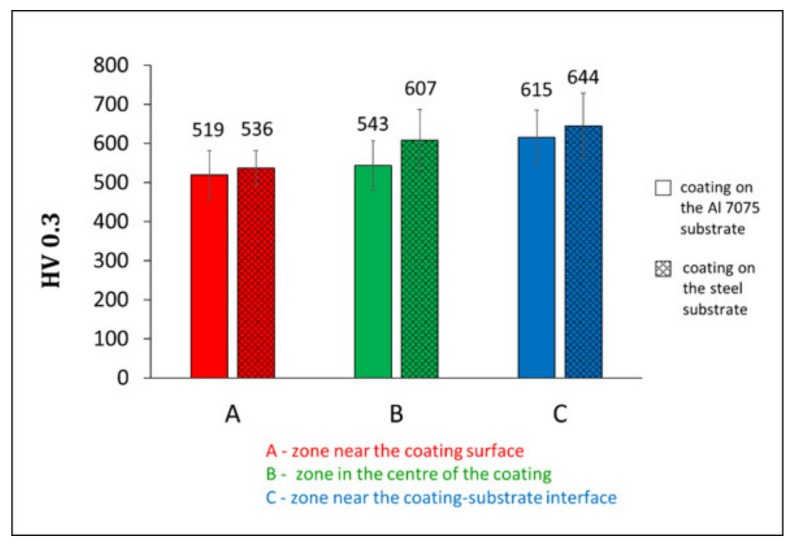
The HV0.3 hardness measured in three zones of cermet coatings deposited on the Al alloy and steel substrates.

The HV0.3 hardness measurements of the (Cr_3_C_2_-25(Ni20Cr))-(Ni25C) coatings were performed in three zones: near the coating surface, in the centre of the deposit, and near the coating–substrate interface. The results presented in Figure 10 showed that the cermet coatings deposited on the Al alloy had a few percent lower microhardness than those on the steel substrate. 

The results corresponded to the higher content of the ceramic phase and lower amount of the graphite built in these deposits and, thus, a greater strain hardening Ni20Cr matrix. This microhardness corresponds with that obtained by Żórawski et al. [19], who investigated the same type of deposits, albeit cold sprayed from the feedstock powder containing a different graphite content, as well as using various spray parameters and working gas combinations. It was also in the range of microhardness (227 HV0.3 to 875 HV0.3) of cold sprayed Cr_3_C_2_-25(Ni20Cr) coatings produced with different parameters of the cold spraying process, but with the use of a powder of the same manufacturer [14,16,17,22]. When analysing the hardness in measurement areas, it was observed that its value increased with the distance from the deposit surface, and the highest was in the zone near the coating–substrate interface, regardless of the substrate material. This was related to the severe deformation strengthening of impact particles in these areas and corresponded with the deposit microstructure, Figure 6.

#### 3.3.2. Flexural Strength of the Coatings

**Figure 11:**
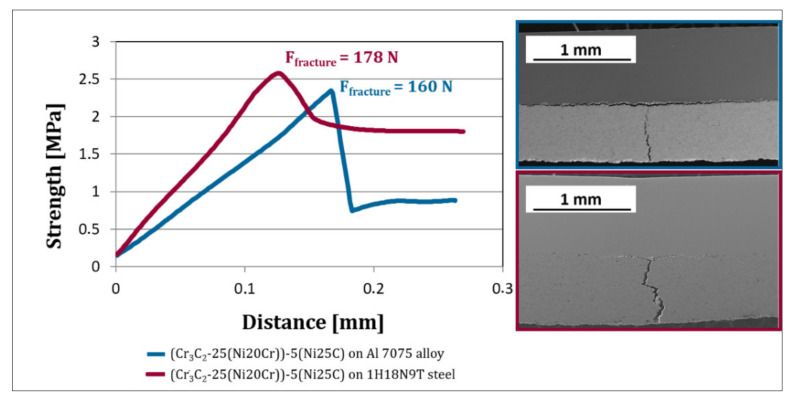
Strength versus displacement of the samples during the three-point bending test and SEM-BSE cross-section microstructures of the coating–substrate systems after the test.

Figure 11 presents the results of the three-point bending tests of the (Cr_3_C_2_-25(Ni20Cr))-(Ni25C) deposits. In both coating–substrate systems, the crack spread along the coating, and the substrate remained undamaged. Moving the former with a constant speed of 0.001 mm/s^−1^ caused an increase in the force acting on the coating–substrate system. Figure 11 shows that, for the coating deposited on the steel substrate, the displacement of the former was ~40% shorter than for the deposit sprayed on the Al alloy. Additionally, the force needed to crack the coating cold sprayed on the steel was 10% higher than that required to destroy the coating deposited on the Al alloy. The determined strength for damage was 2.32 MPa and 2.58 MPa for the coatings deposited on the Al alloy and steel, respectively. The SEM microstructure of the coatings after the three-point bending test showed that one sprayed on the Al alloy revealed a crack more intense in the zone near the substrate than in the deposit, whereas that deposited on the steel substrate showed the fracture larger inside the coating than in the interface zone. This indicates the higher adhesion to the steel substrate.

The substrate affects the three-point bending results of the coating–substrate systems. As presented in Figure 11, the coatings cracked differently. In the deposit deposited on the steel substrate, the crack running perpendicular to the substrate was significantly greater than that localised horizontally near the substrate. Giu et al. [36] and Koutsomichalis et al. [34] observed similar behaviour in the case of WC-Co-Cr composite coatings HVOF sprayed on steel substrates. They showed that cracking of the coating started at the surface and propagated in the deposit towards the substrate. Then, the crack changed direction and spread to a limited extent in the coating–substrate interface and in the coating itself just above the interface [36,37]. In the (Cr_3_C_2_-25(Ni20Cr))-(Ni25C) deposit deposited on the Al alloy substrate, an intense crack was observed in the interface zone, contrary to those sprayed on steel ones. In this case, the fracture in the coating was much bigger than near the substrate. The way the crack proceeds was reflected in the force value required to destroy the durability of the coating–substrate system, which was 160 N and 178 N for the deposit on the Al alloy and steel substrates, respectively. These results correspond to the measurements of adhesion, which showed that the coatings sprayed on the steel substrate revealed a higher adhesion to the substrate than those deposited on the Al alloy. 

#### 3.3.3. Resistance of Wear of the Coatings

**Figure 12:**
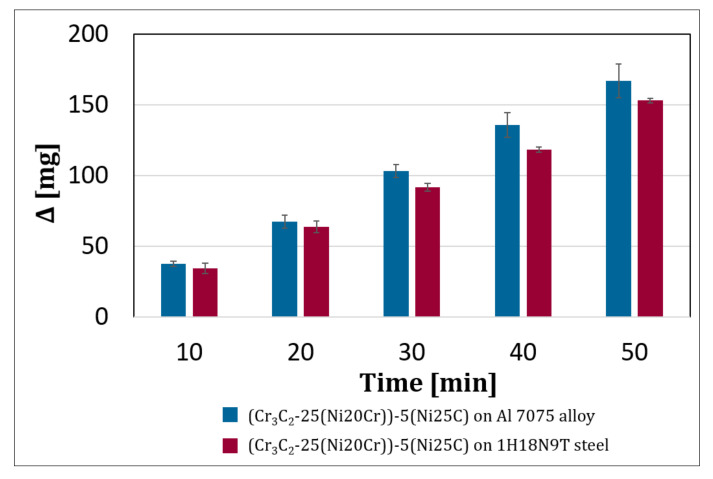
Mass loss versus time during the abrasive wear test.

To check the tribological properties, wear tests with Al_2_O_3_ loose abrasive particles were performed for three (Cr_3_C_2_-25(Ni20Cr))-(Ni25C) coatings deposited both on the Al alloy as well as steel substrates. The loss in coating mass versus time was determined (Figure 12). The tests were carried out in five stages, lasting 10 min each. After the first 20 min, a similar mass loss of the coatings was observed regardless of the substrate used. After 50 min of test duration, the deposits deposited on the steel substrate showed about a 10% lower wear. These results corresponded to the hardness results, and the coating with a higher hardness revealed a lower coating wear. They were also related to the higher content of embedded hard ceramic particles in the deposit cold sprayed on steel.

#### 3.3.4. The Wear Index and the Friction Coefficient

To determine tribological properties, such as the wear index and the friction coefficient, ball-on-disc tests for both coating types were carried out. Table 4 shows the tribological properties of the coatings tested at various temperatures and load conditions. Regardless of the substrate and the temperature used, the wear increased while increasing the load from 10 N to 20 N. Both coating types showed a similar wear index (Wv) under the 20 N load. At 250 °C, the Wv values were almost three times higher than those measured at 25 °C. In addition, the coatings deposited on steel tested at a temperature of 500 °C revealed the best wear properties, wherein those examined under a load of 20 N showed a wear index six times higher than that tested under 10 N. The significantly lower wear was related to changes in their surface layer caused by high temperature and the formation of wear-resistant Cr_2_O_3_ oxide on the surface. This oxide layer significantly improved the wear resistance of the coatings tested under a load of 10 N.

However, their testing under a higher loading force resulted in the removal of oxide and an increase in the wear index. In turn, the friction coefficient determined under a load of 20 N was lower in the coatings deposited on the Al alloy, while, in deposits deposited on steel, it increased with load, except for samples tested at a temperature of 250 °C. The cermet deposit cold sprayed on steel examined at 25 °C under 10 N revealed the best wear resistance and the lowest friction coefficient. The results are similar to those presented by Zhang et al. [38] for plasma sprayed Cr_3_C_2_-NiCr coatings. They tested deposits at the following temperatures: 27 °C, 350 °C, 500 °C, 630 °C, and 750 °C using a 15 N load. The lowest wear rate (~10·10^−6^ mm^3^·N^−1^·s^−1^) and the lowest coefficient of friction (~0.44) were observed for the coatings tested at 500 °C. In turn, Hanyou et al. [39] showed that the MoS_2_ solid lubricant in Cr_3_C_2_-NiCr composite coatings produced by the laser thermal spray method significantly reduced the wear of coatings at room temperature—~40%, and at high temperatures, i.e., 700 °C—~65%. The wear value was 40.31·10^−6^, 31.57·10^−6^, and 31.93·10^−6^ mm^3^·N^−1^·s^−1^ for the Cr_3_C_2_-NiCr coatings tested at 25 °C, 400 °C, and 700 °C, respectively. The Cr_3_C_2_-NiCr+10%Ni/MoS_2_ coatings containing the addition of a solid lubricant were, respectively, 25.97·10^−6^, 30.06·10^−6^, and 11.86·10^−6^ mm^3^·N^−1^·s^−1^.

**Figure 13:**
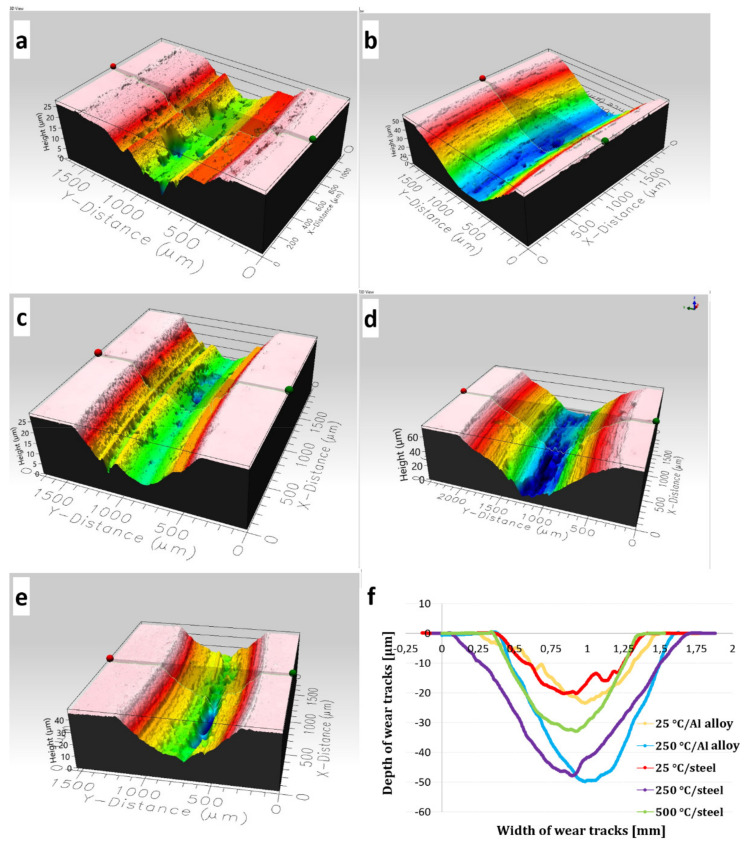
The surface topography of wear tracks of the (Cr_3_C_2_-25(Ni20Cr))-(Ni25C) coatings tested under 20 N load; coating on the Al alloy, 25 °C (**a**), coating on the Al alloy, 250 °C (**b**), coating on steel, 25 °C (**c**), coating on steel, 250 °C (**d**), coating on steel, 500 °C (**e**), and depth vs. width of the deposit wear tracks (**f**).

Figure 13 shows the surface topography of wear tracks, their width and depth determined for the cermet coatings subjected to the ball-on-disc tests carried out with a 20 N load. Figure 13f shows similar values of the wear track depths for coatings tested at 25 °C (~20 µm) and for coatings tested at 250 °C (~50 µm), regardless of the substrate used. The depth of the wear profile of the coating deposited on the steel substrate tested at 500 °C was approximately 30 µm. This proves that fragments of hard Cr_3_C_2_ carbides were torn off during the ball-on-disc test. The above results corresponded to the calculated wear index of the samples presented in Table 4. The coatings tested at 250 °C with a 20 N load had the worst wear resistance (Figure 13). Their wear paths were widest, being 1.2 mm in the coating deposited on the Al alloy and 1.6 mm on the steel substrate. The wear paths of the deposits tested at 25 °C and 500 °C were about 1 mm wide.

The obtained results indicated that the substrate did not affect the wear index and the friction coefficient of the (Cr_3_C_2_-25(Ni20Cr))-(Ni-graphite) cermet coatings, and they can work under frictional conditions. Only the substrate used determines the use of the deposits under specific working conditions.

## 4. Conclusions

The research presents investigations explaining the influence of the substrate material on the microstructure, mechanical, and tribological properties of (Cr_3_C_2_-25(Ni20Cr))-(Ni25C) cermet coatings obtained in the cold spray process. The obtained results are shown below.

1. Both coatings were composed of the same phases as the feedstock powder. The nanostructured grains of the alloying matrix were formed at the particle–particle and coating–substrate interfacial regions during the deposition process, regardless of the type of substrate.
2. Both (Cr_3_C_2_-25(Ni20Cr))-(Ni25C) deposits adhered well to the substrates. However, the coatings sprayed on the Al 7075 alloy substrate showed more ceramic particles stuck onto the substrate than those on the steel.
3. The coating deposited on the 1H18N9T steel substrate revealed a 5% higher hardness and 10% lower abrasive wear with the Al_2_O_3_ loose abrasive particles.
4. The substrate affected the three-point bending results of the coating–substrate systems. In the (Cr_3_C_2_-25(Ni20Cr))-(Ni25C) deposited on steel, the force required to destroy the durability of the coating–steel substrate system was 11% higher than that sprayed on the Al alloy.
5. Both coating types showed a similar wear index (W_v_) under the 20 N load; however, at 250 °C, the W_v_ values were almost three times higher than that measured at 25 °C.
6. The cermet deposit cold sprayed on steel and examined at 25 °C under 10 N revealed the best wear resistance and the lowest friction coefficient.

## Figures and Tables

**Table 1:** The chemical composition (wt.%) of the powders and substrates.

**Diamalloy 3004 (Cr_3_C_2-_25(Ni20Cr))** *(Oerlicon Metco Certificate)*							**Durabrade 2221 (Ni25C)** (*Oerlicon Metco Certificate*)				
**Chemical element**	Ni	C	Others (max)	Cr			Ni	C			
**wt.%**	18.75	9.75	2.25	Balance			75	25			
**Al 7075 alloy** *(EN 573-3:2019(E)) [24]*											
Chemical element	Si	Fe	Cu	Mn	Mg	Cr	Zn	Ti	Others each	Others total	Al
wt.%	0.4	0.5	1.2–2.0	0.3	2.1–2.9	0.18–0.28	5.1–6.1	0.2	0.05	0.15	Balance
**1H18N9T steel** *(EN 10088-3:2014(E)) [25]*											
Chemical element	C	Si	Mn	P	S	Cr	Ni	Others (max)			
wt.%	0.18	1.0	2.0	0.045	0.030	17–19	9–12	Ti:5 × C-70			

**Table 2:** Parameters of the cold-spray process.

Parameters	Values
Working gas	N_2_ + He
Gas pressure [MPa]	4
Temperature [°C]	800
Powder feeder rate [g·mm^−1^]	95 ± 3
Standoff distance [mm]	30
Speed of robot arm [m·s^−1^]	0.3

**Table 3:** The surface topography parameters of coatings according to ISO 25178 [26].

(Cr_3_C_2_-25(Ni20Cr))-(Ni25C) Coatings		Height Parameters						
		S_a_ [µm]	S_q_ [µm]	S_sk_	S_ku_	S_p_ [µm]	S_v_ [µm]	S_z_ [µm]
**Substrate**	**Al 7075**	9.90	12.38	−0.19	3.06	60.88	52.64	113.50
	**1H18N9T steel**	9.60	11.82	−0.14	2.67	43.90	39.79	83.69

**Table 4:** The wear index and friction coefficient of the (Cr_3_C_2_-25(Ni20Cr))-(Ni25C) coatings deposited on different substrates and determined under various conditions.

Coating	Wear Test Conditions		Wear Properties	
	Temperature [°C]	Load [N]	Wear Index, Wv·10^−6^ [mm^3^·N^−1^·s^−1^]	Friction Coefficient
**(Cr_3_C_2_-25(Ni20Cr))-(Ni25C) on Al 7075 substrate**	25	10	18.3 ± 8.5	0.76 ± 0.03
		20	28.7 ± 5.8	0.70 ± 0.02
	250	10	38.2 ± 2.1	0.57 ± 0.02
		20	86.0 ± 15.6	0.50 ± 0.01
**(Cr_3_C_2_-25(Ni20Cr))-(Ni25C) on 1H18N9T steel substrate**	25	10	23.5 ± 4.1	0.65 ± 0.07
		20	29.3 ± 7.4	0.70 ± 0.01
	250	10	45.3 ± 3.4	0.59 ± 0.01
		20	85.0 ± 12.5	0.56 ± 0.01
	500	10	11.4 ± 2.8	0.36 ± 0.01
		20	65.3 ± 29.6	0.40 ± 0.01

## Data Availability

Data sharing not applicable.
